# It takes a village: how plant-associated bacterial communities socialise to shape plant growth and health

**DOI:** 10.1042/EBC20250038

**Published:** 2026-09-02

**Authors:** Carmen Escudero-Martínez, Andrzej Tkacz, Marta Albareda, Carmen Sánchez-Cañizares

**Affiliations:** 1 Consejo Superior de Investigaciones Científicas, Instituto de Recursos Naturales y Agrobiología de Salamanca (IRNASA-CSIC), Salamanca 37008, Spain; 2 Centro de Ciências do Mar do Algarve (CCMAR/CIMAR LA), Universidade do Algarve, Campus de Gambelas, Faro 8005-139, Portugal; 3 Centro de Biotecnología y Genómica de Plantas, Universidad Politécnica de Madrid-Instituto Nacional de Investigación y Tecnología Agraria y Alimentaria (INIA/CSIC), Madrid 28223, Spain; 4 Departamento de Biotecnología-Biología Vegetal, Universidad Politecnica de Madrid, Escuela Tecnica Superior de Ingenieria Agronomica Alimentaria y de Biosistemas, Madrid 28040, Spain

**Keywords:** beneficial bacteria, bioinoculants, plant growth promotion, plant holobiont, plant-associated bacteria, plant-microbe interactions

## Abstract

Plants outsource many functions to an associated microbiome, an ‘outside gut’ composed of socially interacting bacterial communities that support plant growth and health. These dynamic and diverse microbiomes span different plant microenvironments and form complex networks shaped by the environmental context and the plant’s genetics. While some microbes cause disease, many others contribute to essential functions, influencing plant fitness, survival, and ultimately soil health. Although the host genetics and abiotic factors structure these communities, the functional diversity and ecological roles of plant-associated bacteria are ultimately shaped by microbe–microbe interactions. These interactions also play a critical role in the successful colonisation of plant tissues. Beneficial members of these communities enhance plant nutrient uptake, modulate phytohormones to shape root architecture, or prime plant systemic immunity to repel pathogens. Deciphering how plant-associated bacterial communities assemble and interact, both with their host and with one another, is essential for uncovering the mechanisms that underpin bacterial social behaviours within the plant holobiont, such as quorum sensing, metabolite exchange, and contact-dependent interactions. Here we review several recent publications that shed new light on the social lives of plant-associated bacteria, with a particular focus on their competition mechanisms and strategies for colonisation and establishment. In this context, we highlight how the rational design of plant bioinoculants based on microbial consortia represents a powerful strategy for promoting sustainable agriculture in a rapidly changing environment.

## Introduction

A plant together with all its associated microorganisms functioning as a single biological, ecological, and evolutionary unit is known as the plant holobiont [1,2]. Members of plant-associated microbial communities may colonise intra- or intercellular spaces, as well as the surfaces of aerial and belowground organs [3,4]. These interactions can be explained by the fact that plants, as autotrophic organisms, fix atmospheric carbon and represent a source of nutrients for microbial communities. This, in turn, is a driver for plant colonisation and competition with other microbes for the plant resources. On the plant side, there is a selection for microbial traits that provide a functional advantage to the plant, for example, access to soil-bound nutrients or pathogen protection [5,6]. Other microbes can also act as commensals, indirectly supporting such microbial traits. Indeed, many beneficial microbial traits, like pathogen protection in suppressive soils, cannot be explained by a single microbe but at the microbial community level [7,8]. Thus, the host plant imposes selection on its associated microbial communities, shaping their composition and functional potential [9]. Among plant-associated microbes, bacteria dominate in both abundance and diversity, but only those strains possessing traits that enable effective competition, colonisation, and survival in the plant environment can establish associations with their host, while others remain confined to the surrounding soil [10]. Taxonomically, the most common plant-associated bacteria belong to four phyla, Pseudomonadota, Actinomycetota, Bacteroidota, and Bacillota (previously named Proteobacteria, Actinobacteria, Bacteroidetes, and Firmicutes) [9]. Therefore, with a focus on plant-associated bacterial communities, i.e., the plant-associated bacteriome [11], the present review highlights key plant and bacterial determinants of successful association with their plant hosts, including bacteria–bacteria interactions, their spatial organisation, and the roles of environmental factors such as pH. These interactions unfold differently in the field than under tightly controlled laboratory cultures, with consequences for plant growth and health. All of these interactions have implications for proper ecosystem functioning, a requisite for sustainable crop production. While we focus on bacterial mechanisms, it is important to recognise that bacterial interactions are embedded within complex interkingdom networks involving fungi, archaea, protists, oomycetes, viruses, and host organisms, all of which contribute collectively to the holobiont framework, and more broadly, ecosystem function. Throughout the present review, we use terms such as *social behaviour, social interactions*, or *socialisation* to refer to collective interactions that emerge from molecular mechanisms that alter the physiology or fitness of neighbouring cells.

## Factors involved in a successful bacterial colonisation and establishment

**Figure 1:**
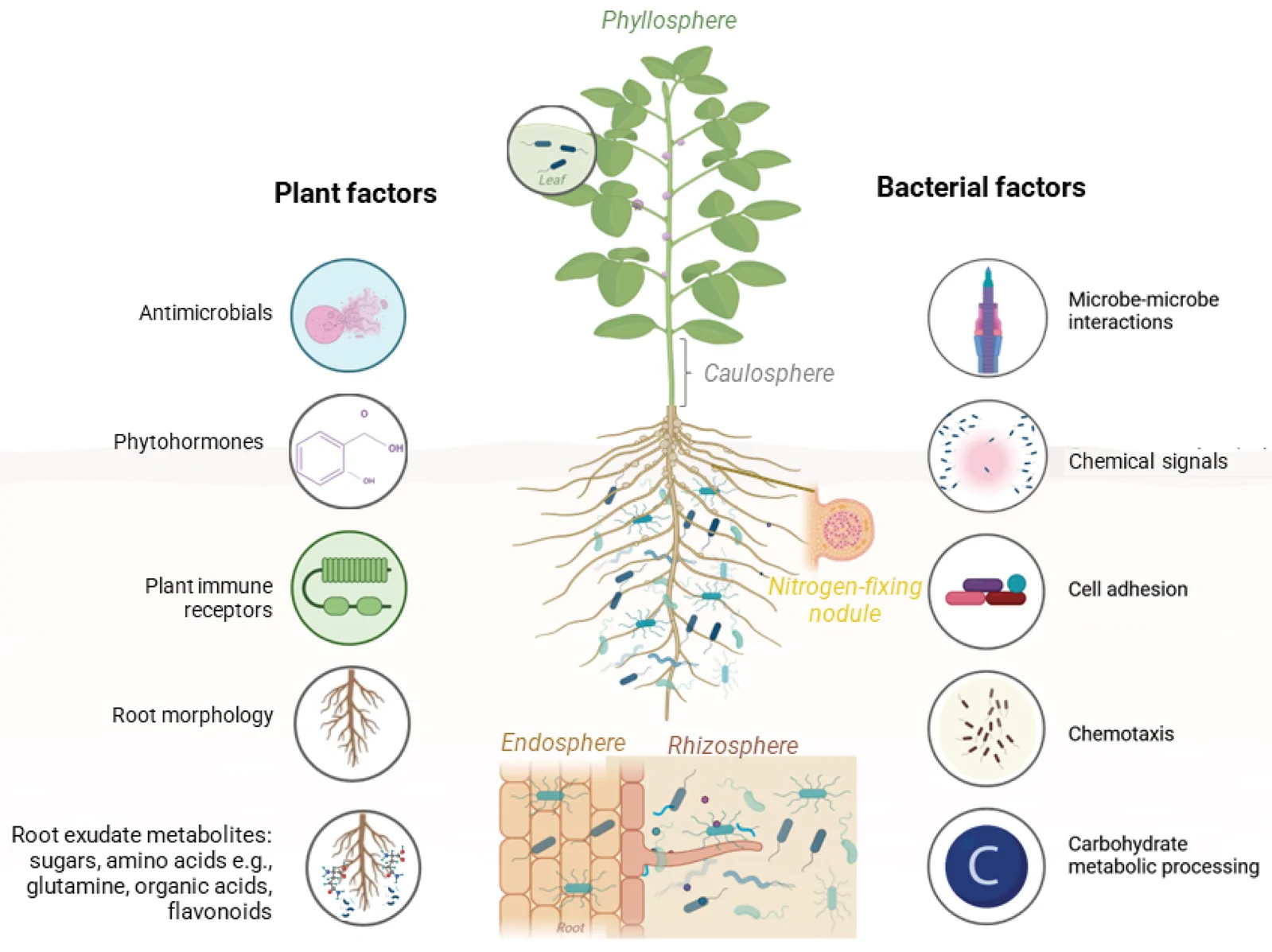
Overview of plant and bacterial determinants shaping plant–bacteria interactions Plant factors include root morphology, root exudate composition, immune signalling pathways, phytohormones, and antimicrobials. Bacterial factors encompass colonisation traits such as carbohydrate metabolism, chemotaxis, cell adhesion to plant surfaces, responses to chemical signals, and microbe–microbe interactions. Selective pressures between the host plant and bacterial functional attributes determine community assembly, stability, and bacterial traits related to plant health and nutrition. Figure created with BioRender and BioIcons.

Plants actively shape their rhizosphere (i.e., the interface between roots and soil) microbiome to their own benefit, effectively exploiting its functional repertoire [12,13]. The bulk soil is a microbial reservoir from which plants select a specific subset of associated microbes [14]. Plant features such as root exudation profiles, immune signalling, or morphology (Figure 1) drive this microbial assembly [15]. Such features vary according to the plant organ, differentiating microbial niches within the same plant [14]. The plant species [16], the genotype [17–19], and the developmental stage [20] further structure the plant-associated microbiota. Indeed, correlations have been found between plant phylogenetic distance and root-associated microbiota dissimilarity [21–23]. Plant genomes are organised at multiple levels, including epigenetic modifications, transcription factors, and regulatory RNAs, which collectively control plant trait plasticity for adaptation to a changing environment. This flexibility may promote a dynamic plant-associated microbiome [24,25].

Plants release photosynthesis-derived carbon in the form of root exudates into the rhizosphere [26]. This ability is one of the most remarkable metabolic features of plant roots, with nearly 5% to 21% of all photosynthetically fixed carbon being transferred to the rhizosphere through root exudates [27,28]. Driven by this carbon deposition around the roots, the total abundance of bacteria increases in the rhizosphere relative to the surrounding unplanted soil [9,29]. In addition to carbohydrates and amino acids, which act as primary chemical metabolites in the rhizosphere [30], plant secondary metabolites (PSMs) are also key drivers in the development of plant-specific microbial communities [31–34]. Another driver is the plant immune system, known to modulate microbial assembly and colonisation via phytohormones like salicylic acid (SA) [35] or jasmonic acid (JA) [36]. *Arabidopsis* isogenic mutants that produce and store enhanced levels of SA increased the endosphere abundance of Proteobacteria, while plants impaired in SA synthesis showed increased abundance of Bacteroidetes in root tissue [35].

Plants typically shape microbial communities through controlled exudation via membrane transporters or root cap cell shedding [15], yet endodermal leakages provide a third, less selective route that releases low-molecular-weight metabolites into the rhizosphere. These leakages seed microbial communities at micrometre scales, where competition and cooperation for valuable metabolites unfold. Plants exert their control over their microbial partners at different levels, with the Casparian strip emerging as a central regulator of root microbial recruitment and community composition [37]. This lignin-rich endodermal barrier normally restricts solute diffusion from the vasculature, but at natural breaks or incomplete zones, transient glutamine leakages create short-lived, spatially restricted metabolic niches that attract soil bacteria [38]. Glutamine is thought to be the main intracellular signal for nitrogen availability in most bacteria [39], enabling bacterial cells to alter their physiology through complex regulatory systems [40–42]. Tsai et al. [38] reported how glutamine leaked by the plant acts both as a nutrient and a chemoattractant, guiding the colonisation of the plant-growth-promoting (PGP) rhizobacterium *Pseudomonas protegens* CHA0. This process is particularly pronounced near lateral root emergence sites and early differentiation zones, where the Casparian strip is not continuous. Casparian strip-deficient *Arabidopsis thaliana* mutants exhibited excessive bacterial proliferation, demonstrating that nutrient restriction is essential to prevent bacterial over-colonisation that could compromise plant health. In legumes, the Casparian strip inside nodules ensures a balanced nutrient exchange for an effective symbiosis [43]. Upon inoculation with the symbiont *Mesorhizobium loti* R7A, *Lotus japonicus* mutants with defective barriers showed impaired nodulation due to disrupted root-to-shoot signalling, resulting in reduced infection threads, smaller nodules, and consequently decreased nitrogen fixation [43]. These findings offer a plausible explanation for the plant’s metabolic investment in amino acid exudation as a cue for bacterial colonisation, and reveal an additional layer of control over its microbial partnerships.

**Figure 2:**
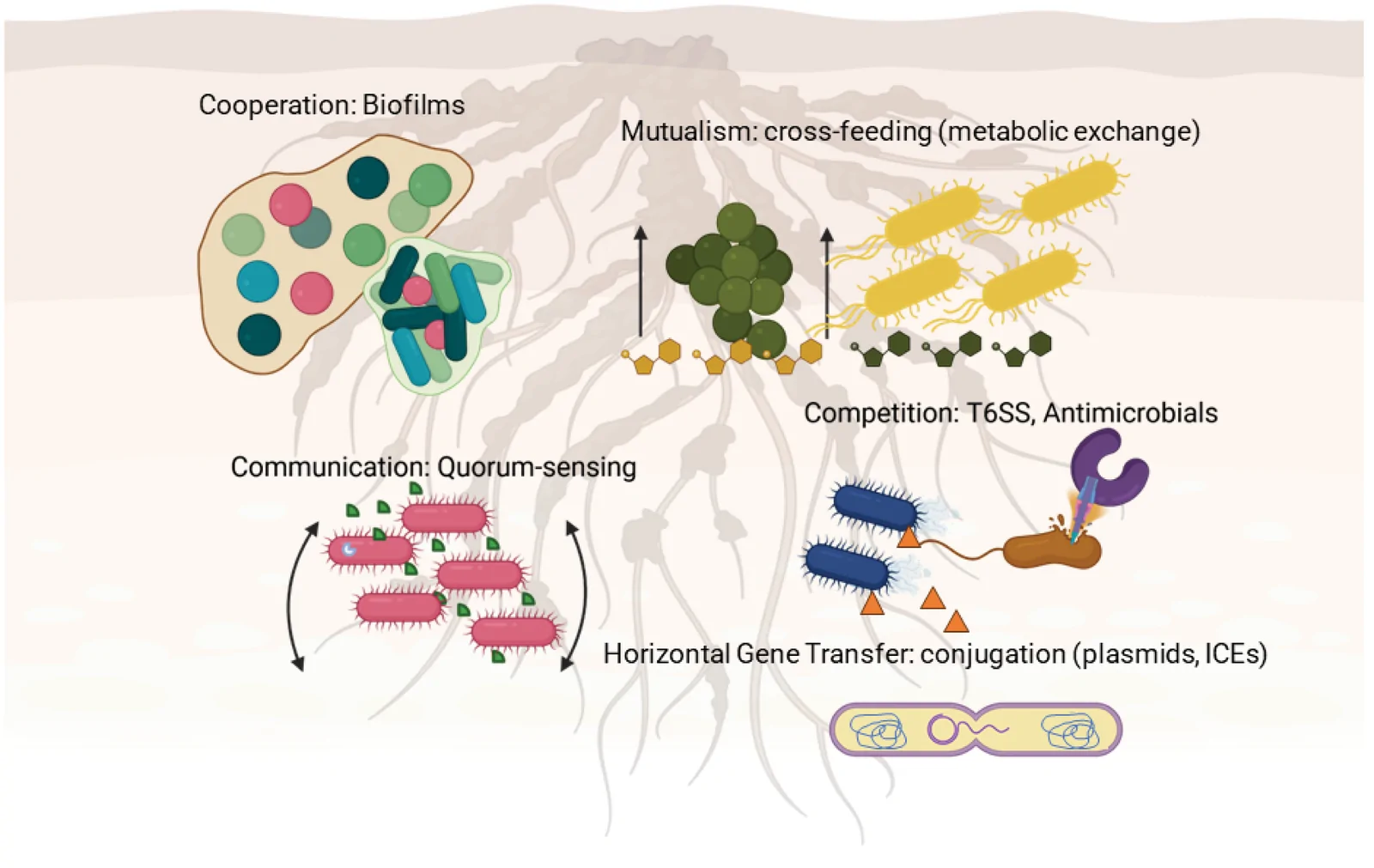
Conceptual representation of ‘*The social lives of plant-associated bacteria*’ related to bacteria–bacteria interactions within plant-associated bacteriomes Cooperative processes include biofilm formation, metabolic cross-feeding, mutualistic exchanges, and quorum sensing-mediated communication, which enhance community stability and functional integration. Competitive interactions involve resource competition, secretion of antimicrobials, and contact-dependent antagonism such as type VI secretion systems (T6SS), shaping niche partitioning and exclusion dynamics. Horizontal gene transfer (HGT) further contributes to community adaptation and evolution by spreading functional traits *via* bacteria–bacteria conjugation of plasmids or integrative conjugative elements (ICEs). Figure created with BioRender and BioIcons.

Successful microbial plant colonisers share conserved functional traits for competing against other organisms, accessing, and exploiting plant-derived resources [44]. The metabolic potential of rhizosphere bacterial communities, compared with that of unplanted soil, has been described as enriched in gene ontologies related to carbohydrate metabolic processing, cell adhesion, pathogenesis, response to abiotic stimulus, responses to chemicals, and cell motility (Figure 2) [45]. Accordingly, a pioneering Tn5 mutant screening on *Pseudomonas fluorescens* WCS365 identified efficient chemotaxis systems and rapid utilisation of low-molecular-weight carbon compounds as central traits for root-associated bacteria [46]. Likewise, a comparative genomics study of 484 bacterial isolates from *Brassicaceae* roots identified an enrichment of genes involved in carbohydrate metabolism and a depletion of mobile elements. These genomic features included genes associated with plant colonisation, microbe–microbe interactions, and host interactions, such as mechanisms involved in modulating host immunity, including secretion systems or effector proteins [47].

Microorganisms are capable of colonising previously underexplored plant-associated niches, like plant stems or flowers, where they display distinct adaptive traits. At a larger plant scale, metagenomic analyses in Australian forests have shown that tree bark hosts unexpectedly dense and diverse endophytic microbial communities that are metabolically distinct from those in surrounding soils and freshwater wetlands [48]. These bark-associated microbes, known as the caulosphere, possess remarkable metabolic flexibility, enabling them to consume or produce climate-active gases such as methane (CH_4_), hydrogen (H_2_), carbon monoxide (CO), carbon dioxide (CO_2_), and volatile organic compounds. Their activities substantially influence atmospheric gas fluxes, with bark communities consuming CH_4_, H_2_, and CO at rates comparable to soils. These findings challenge the long-held assumption that soils dominate trace gas exchange and reveal that bark, covering an estimated surface of ∼143 million km^2^ globally and previously considered nutrient-poor for sustaining microbial life, plays a prominent role in regulating biogeochemical cycles. The metabolic versatility of its associated microbiota suggests they may contribute to climate mitigation, making the incorporation of bark microbiomes into climate models and forest management strategies essential.

## The benefits of engaging a diverse bacterial community

Maintaining microbial diversity is key for plant resilience and survival. For instance, avoiding pathogen outbreaks has often been related to microbial imbalances and low diversity, termed ‘dysbiosis’ [49]. As with any social system, the strength and stability of plant-associated microbial communities depend not only on the nature of the interaction (beneficial or detrimental) but, more importantly, on the diversity of the microbial players involved. A heterogeneous bacterial community may enhance the genetic potential and functional capacity, providing the foundation upon which cooperative and competitive interactions unfold, ultimately influencing plant performance. Using cultivable maize root isolates, Niu et al. [50] assembled a seven-member synthetic community (SynCom) and tested how this simplified consortium assembled and functioned within maize roots. In ‘dropout’ experiments (removing one strain at a time) done in laboratory flasks, the community showed robust stability, except when *Enterobacter cloacae* (now renamed *Enterobacter ludwigii*, Gammaproteobacteria) was absent. This absence caused a major community restructuring in which *Curtobacterium pusillum* (Actinobacteria) rose to dominance. This suggests a keystone (i.e., a species driving key ecological processes despite occurring at low abundance [51]) and stabilising role for *Enterobacter cloacae*. It also illustrates that simplified root consortia (such as this SynCom) can help reveal keystone members more easily, as the functional redundancy typical of complex natural microbiomes is reduced. Functionally, this SynCom suppressed the fungal pathogen *Fusarium verticillioides in planta* and *in vitro*, linking bacterial community structure to host protection. Yet, the mechanism(s) underlying fungal suppression and community stability were not resolved. Plausible explanations might include antimicrobial production (e.g., bacteriocins) or interference competition, where some bacterial strains inhibit other microbes (e.g., via Type VI secretion system, a contact-dependent weapon that enables bacteria to inject antibacterial effectors into neighbouring bacterial cells (Figure 2) [52–55]).

However, plant-associated habitats are not flask environments: roots and soils are spatially structured, and this can profoundly alter the bacterial interactions and ‘wipe-out’ dynamics (i.e., removal of a given species) observed when cells are well-mixed in laboratory cultures. This is precisely the point demonstrated by microspatial ecology experiments [56]. By placing microbial competitors into defined arrangements at micron scales using a droplet printing method, Kumar et al. showed that outcomes of toxin-mediated competition (i.e., colicins in *Escherichia coli*) can differ depending on whether strains are mixed or segregated [57]. In mixed cultures (i.e., shaking flasks), toxin producers efficiently eliminated susceptible neighbours. By contrast, when bacterial populations were arranged in an organised pattern, susceptible cells could persist in an ecological refuge even though the toxin could diffuse. This refuge emerges because cells at the ‘front line’ absorb the toxin, shielding those behind, and transforming susceptibility into a collective, spatially mediated protection rather than a purely individual vulnerability. Such a ‘front line’ pattern can also be observed on plant roots, where bacteria adhere to root surfaces and organise into layered biofilms (i.e., a matrix formed primarily of extracellular polysaccharides that enhances stability, communication, and resource exchange, Figure 2) [58,59]. Translated back to Niu’s ‘flask environment experiment’ [50], this suggests that even if *Curtobacterium* (or another member of the maize root SynCom) produces inhibitory compounds, effective exclusion may be limited by patchiness, microscale diffusion constraints, and neighbour shielding, allowing competitors to persist in protected pockets on the root surface, within biofilms, or in soil aggregates. Overall, these strategies allow the maintenance of microscale microbial reservoirs that can act as sources of functional microbial diversity under favourable conditions.

Another important determinant of bacterial ‘social’ interactions arises from microbial environmental engineering [60]. Ratzke and Gore [61] showed that many bacteria shift environmental pH as a by-product of their metabolism and that this growth–pH feedback can generate predictable microbial interaction responses without involving specialised signalling. In laboratory cultures, a pH shift may lead to ecological suicide: a bacterium can acidify its environment beyond survivable limits. However, such dramatic effects are unlikely to occur in natural environments, where multiple buffering processes operate [62]. In plant systems, this framework maps onto root chemistry. Through chemotaxis-driven motility, bacteria detect gradients of root exudates and actively migrate towards them, a key step that precedes root surface attachment and successful colonisation [63,64]. These carbon-derived compounds feature a large diversity in composition and ecological roles, acting as signalling molecules, attractants and/or repellents, and, importantly, as nutrients for microorganisms [65], representing one of the main drivers of microbiota assembly in plants [66,67]. Different root exudate profiles (e.g., organic acids, sugars, amino acids) can favour metabolic pathways that either acidify or alkalinise the rhizosphere, enabling some taxa, while pushing others outside their tolerated pH ranges. The ability of some rhizosphere bacteria to modify or tolerate local pH fluctuations might provide a competitive advantage during root colonisation. Bacteria capable of buffering or withstanding metabolically induced pH shifts may maintain active metabolism and continue importing root exudate-derived nutrients under conditions that impair the growth of less tolerant taxa. In contrast, neighbouring microbes experiencing unfavourable pH conditions may suffer metabolically stressed states, reducing their capacity to exploit available carbon sources. Such dynamics could create transient competitive windows in which pH-resilient taxa gain disproportionate access to exudate nutrients, influencing microbial community assembly around plant roots. These effects may also extend to endophytic niches inside plant tissues (i.e., endosphere), where buffering capacity and resource availability drastically differ from those of the rhizosphere. Importantly, bacteria possess a cell-to-cell communication mechanism known as quorum sensing (Figure 2) that uses small chemical molecules to detect population density, enabling plant-associated bacteria to coordinate gene expression and synchronously alter their behaviour in processes such as motility, biofilm formation, root colonisation, or metabolite production, influencing plant growth and health [68–70]. Recent work in *Arabidopsis* and *Pseudomonas aeruginosa* has demonstrated that plants can intercept bacterial quorum sensing signals to mount a coordinated, pre-emptive defence involving immune activation, chemical disruption of bacterial communication, and microbiome reconfiguration, highlighting the bidirectional nature of interkingdom signalling [71].

Together, these studies suggest that community-level behaviours (such as pathogen suppression) can be robust and reproducible, and that the underlying bacterial ‘social’ (i.e., microbe–microbe) interactions may arise from multiple factors, such as (i) antagonism constrained by spatial refuges and/or (ii) metabolic pH engineering and feedback, both of which are amplified, rather than simplified, by the structured, heterogeneous root habitat.

## Bacteria shape plant growth and health

When beneficial microbial communities establish mutualistic associations with plants, they enhance plant performance (e.g., by producing growth-promoting hormones like auxins or cytokinins [72,73]), influence its development (e.g., flowering time or root growth [74,75]), impact nutrient uptake (e.g., through nitrogen fixation or phosphate solubilisation [5,76,77]), and improve plant health by inducing resilience to biotic and abiotic stresses [78,79]. These interactions are shaped not only by individual microbes but also by whole-community dynamics, with plants actively recruiting microbes under specific conditions. For decades, beneficial microbes such as rhizobia, mycorrhizae, and PGP rhizobacteria have been applied to strengthen plant nutrition for sustainable agriculture. In the case of rhizobia, this endosymbiotic relationship strongly shapes the assembly of the legume root microbiota, and its effects depend on the plant’s nitrogen nutritional state. Tao et al. [80] have shown that in *L. japonicus*, different nitrogen sources and nitrogen regimes (nitrogen-starved, symbiotic nitrogen, and nitrate-replete) impact the root and rhizosphere microbiomes, differing markedly in composition and expected bacterial genera. However, the opposite effect was observed in *Medicago truncatula* [81], where the microbiome assembly was only weakly dependent on the soil nutrient level. This suggests an effect of the soil or the lab-specific conditions, but also that some bacteria may simply not strongly respond to nitrogen. Nitrate fertilisation induces root-associated microbiomes distinct from those of nitrogen-fixing plants. Indeed, a meta-analysis based on 908 field observations determined that nitrogen enrichment reduces symbiotic nitrogen fixation by about one-third [82]. Indeed, plants sense nitrate-driven zinc gradients levels and adjust legume nodulation accordingly [83]. Differences in the plant host physiology (including metabolic states and root exudate profiles) further shape the microbial composition, predicting the nitrogen source. A comprehensive assessment of the common bean rhizosphere microbiome after PGP rhizobacterial inoculation showed increased rhizosphere diversity and changes in interactions among different taxonomic domains [84]. However, inoculation of *Medicago truncatula* with highly efficient endosymbionts was associated with reduced root endosphere bacterial diversity across soils, suggesting more selective recruitment of the endosphere microbiome [85]. Overall, these results show that the nitrogen regime, symbiotic signalling, and host genetic background jointly orchestrate the root microbiome assembly, and that interactions between the host and specific symbionts can influence the behaviour and composition of the broader microbial community.

In terms of plant’s health, the plant’s microbial partners are known as ‘the extended plant immune system’ [86]. This concept considers both the host and its associated microbiota, which interact as a coordinated community to avoid pathogen dominance through multiple and complementary strategies. These include the activation of plant immune responses (e.g., induced systemic resistance, ISR), the selective recruitment of beneficial microbes over harmful ones, or the direct suppression of pathogens via biocontrol. Plants recruit protective microbes triggering ISR, expanding their defensive capacity. In turn, activation of defence-related hormones reinforces these associations. For instance, the activation of JA signalling through methyl jasmonate application in the *A. thaliana* rhizosphere increased the abundance of bacterial taxa linked to plant defence, such as *Bacillus* and *Paenibacillus* [36]. Plants also shape their microbiota by discriminating between beneficial and detrimental taxa. Coumarins, a class of phenolic specialised metabolites found in root exudates, exemplify this dual role, as they promote ISR-eliciting PGP rhizobacteria while restricting undesirable microbes [87–89]. Certain legumes produce nodule cysteine-rich (NCR) peptides that define engagement in the legume–*Rhizobium* symbiosis [90–92], controlling the bacterial development inside root nodules. In exchange, bacterial modifier enzymes like HrrP, a host range restriction peptidase capable of degrading NCR peptides, confer more opportunistic behaviour on rhizobia, leading to a suppression of nitrogen fixation [93]. Finally, microbiota-mediated protection can also occur through direct antagonism of pathogens. For example, *Pseudomonas aeruginosa, Trichoderma harzianum*, and *Bacillus subtilis* have been used as biocontrol agents in pea plants [94]. Plants treated with this consortium and challenged with the fungal pathogen *Sclerotinia sclerotiorum* under greenhouse and field conditions showed enhanced growth and reduced disease symptoms.

Bypassing the plant immune system is a prerequisite for establishing a bacterial association with the host plant. Components of the two-tier plant immune system, the pattern-recognition-receptors (PRRs) and, particularly, nucleotide-binding leucine-rich repeat (NLR) proteins are linked to changes in the microbiota composition [95]. Nevertheless, plants have been shown to prioritise nutritional stress over defence; under high phosphate, *Arabidopsis* plants maintain stronger immunity and interact less with microbes, whereas phosphate limitation promotes microbial engagement [96]. Regarding PRRs, studies using single-gene *Arabidopsis* mutants defective in the well-known receptors *fls2, efr, lore*, and *lik4*, concluded that the effects on overall microbiome composition were minor [97]. This may be due to conserved patterns shared between pathogens and commensals, as well as the capacity of the latter to suppress in many instances PRR recognition [98]. In contrast, other PRRs such as LysM were found to be important for maintaining well-known symbiont families, Rhizobiaceae and Burkholderiaceae, in the rhizosphere of *L. japonicus* [80]. Diverse intracellular NLRs were discovered by plant genetic screens related to changes in the microbiota composition [17,99,100]. One such example is the *Arabidopsis* Toll/interleukin-1 receptor (TIR-NLRs or TNLs). This immune receptor was identified by measuring the growth responses of 410 *Arabidopsis* accessions grown on two benzoxazinoids preconditioned soil microbiomes to study microbiome-mediated soil feedbacks to plants [100].

When trying to better understand these beneficial traits, studying microbes individually cannot capture their context-dependent effects within diverse communities. To analyse whole-community properties, Emmenegger et al*.* [101] used axenic *A*. *thaliana* Col-0 plants and 136 SynComs with five members each. They evaluated four microbiota features (i.e., absolute commensal colonisation, community evenness, phylogenetic diversity, and strain identity) for their ability to predict plant protection against the foliar pathogen *Pseudomonas syringae* DC3000. They showed how certain leaf (i.e., phyllosphere) communities strongly reduced pathogen colonisation, in contrast with axenic infected controls. Machine-learning analyses revealed strain identity as the strongest ‘plant protection’ predictor, highlighting *Acidovorax* Leaf76, *Rhizobium* Leaf68, and *Pseudomonas* Leaf15 as ‘pathogen-reducing strains’. Experimental validation confirmed that they reduced pathogen colonisation by two orders of magnitude when tested individually, with additional synergistic effects further enhancing protection by one order of magnitude in binary combinations and in the full combination, suggesting complementary mechanisms for reducing pathogen colonisation.

## Challenges and methodologies in plant bacteriome assemblies for agriculture

In agriculture, bioinoculants based on beneficial plant-associated microorganisms promote sustainable crop production while reducing reliance on agrichemicals [102]. Although they are used successfully worldwide [103], inconsistent or poor field performance has been reported in multiple scenarios. These limitations are primarily attributed to insufficient microbial establishment or plant colonisation, the influence of environmental factors (e.g., soil conditions differing from those of the original isolation site), competition with indigenous microbiota, and plant genotype-dependent selection [104–109]. When plants are grown in new locations, they encounter distinct soil microbial communities, and one possible outcome is disease outbreak due to the loss of protective microbiota and altered plant–soil feedbacks that need to be re-established [110,111]. In legumes, their introduction into novel soil environments is particularly challenging because they may not encounter their compatible nitrogen-fixing symbionts [112]. As a result, effective cultivation often requires inoculation with appropriate rhizobia, typically isolated from nodules of plants grown in their native environments [113–115]. HGT events, which play a crucial role in microbe–microbe interactions (Figure 2), are key for the effectiveness of plant bioinoculants [116]. This microbial mechanism enables microbes to rapidly exchange functional traits that are usually located in their accessory genome (i.e., nutrient acquisition, stress tolerance, or antimicrobial production), reshaping community dynamics while enhancing the adaptive potential of strains in the rhizosphere [117–119].

**Figure 3:**
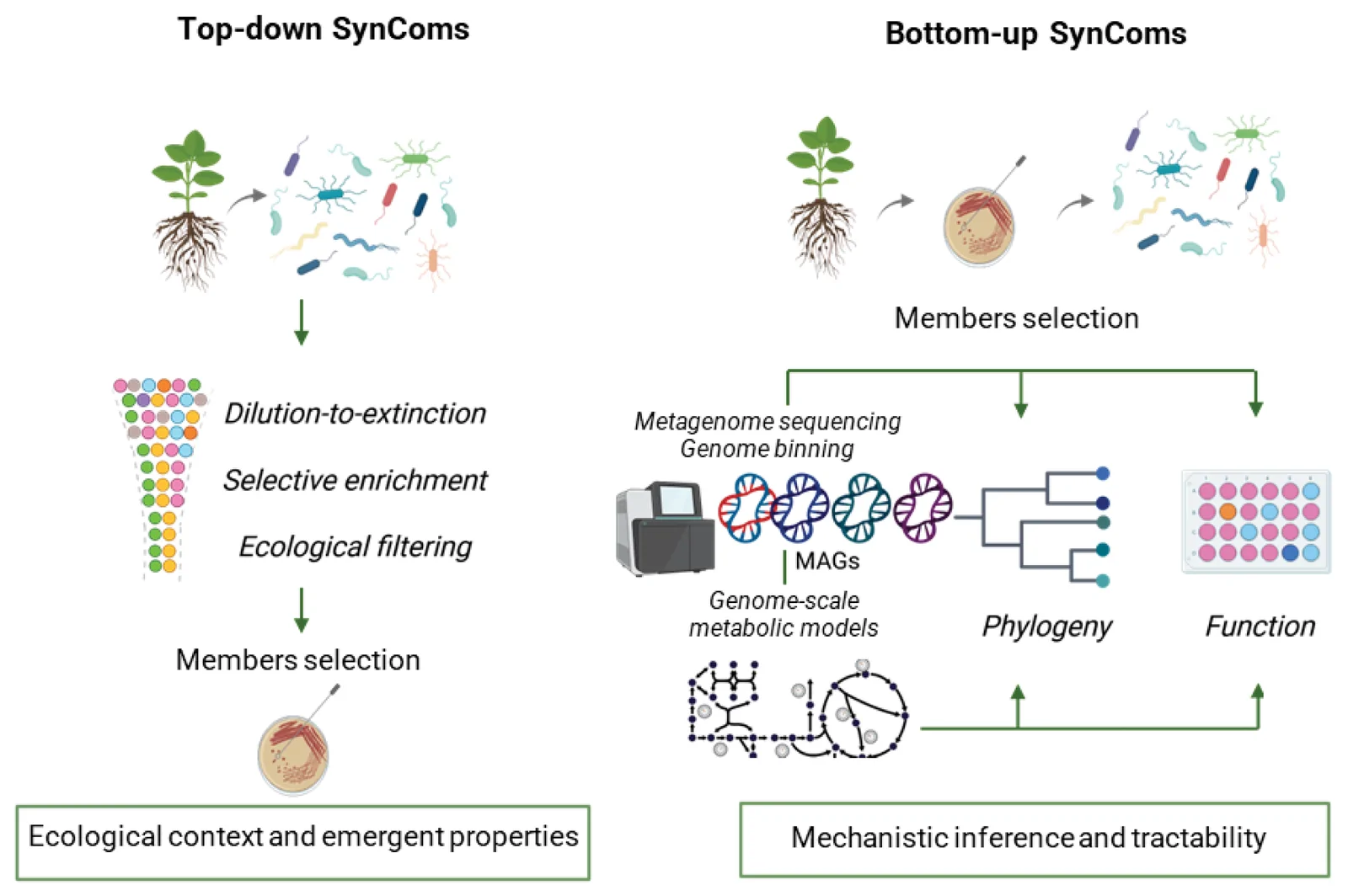
Strategies for SynCom design Top-down assembly (Top-Down SynComs) starts from complex natural microbiomes and progressively simplifies them through dilution-to-extinction, selective enrichment, or ecological filtering to retain desired functions while reducing complexity. Bottom-up assembly (Bottom-up SynComs) constructs communities from isolated strains chosen for defined traits, then tests their collective performance under controlled conditions. This reductionist strategy aims to infer causal relationships and emergent functions from known components. Top-down approaches preserve ecological realism and emergent properties shaped by their native context and priority effects, whereas bottom-up approaches emphasize mechanistic inference and tractability. Figure created in BioRender.

In legumes, strategies based on the use of highly effective and competitive rhizobial strains adapted to local conditions have resulted in plant positive responses to the inoculation [120,121]. However, locally adapted competitive microbial inoculants requiring site-tailored formulations may not be of interest to industry, which generally designs broad-spectrum inoculants intended for multiple crops and soil types. In this regard, designing inoculants considering rhizobial × plant genotype combinations improves symbiotic efficiency and strain competitiveness [122]. Similarly, the plant genotype further modulates other PGP bacterial inoculation outcomes [123]. This indicates the need for co-optimisation of microbial strains and host plant genetic backgrounds. Nevertheless, additional considerations arising from interactions between symbiotic partners and site-specific environmental factors must be considered [124]. Conventional bioinoculants have typically relied on single bacterial strains, an approach that overlooks the synergistic functions of microbial communities and likely explains the variable efficiency of many commercial inoculants [104]. Current insights into plant–microbe interactions pave the way for plant and microbiome engineering approaches aimed at shaping plant-associated communities towards achieving specific plant traits [125,126]. Innovative approaches increasingly use engineered signalling molecules to specifically recruit beneficial microorganisms. Rhizopines (i.e., inositol derivatives of bacterial origin [127]) are a key example of this technology, being engineered to be produced by the host and metabolised only by specific and narrow-range rhizobia, selectively enriching bacteria capable of utilising them [128]. Advances in next-generation sequencing and omics approaches are also expanding the taxonomic diversity and functional breadth of plant-associated microbiomes and their potential as inoculants [129]. This knowledge has led to the design of artificial microbial consortia, or SynComs, as a strategy to mimic the structure and function of the plant microbiome, aiming to overcome field performance limitations while contributing to plant growth and health [130,131]. Two complementary strategies are generally used to design SynComs: top-down or bottom-up assemblies (Figure 3).

Top-down assemblies (Top-Down SynComs) begin with complex natural microbiomes and systematically simplify them, using dilution-to-extinction, selective enrichment, or ecological filtering, to retain target functions while reducing community complexity (Figure 3). One example of this approach is the research of Goldford et al. [132] in which SynComs were derived from both diluted native soil and plant leaves (phyllosphere) bacterial communities that were grown on glucose as the sole carbon source. The resulting community was then diluted and maintained via metabolic cross-feeding, leading to no competition for resources. Repeated dilution cycles reduced initial diversity to a five-member community dominated by Enterobacteriaceae and Pseudomonadaceae. Across 12 independent inoculations, communities consistently converged at the family level but were distinct at lower taxonomic ranks. This indicates that the constrained growth conditions enriched recurrent metabolically compatible (non-competing) taxa. Another example of top-down selection is the enrichment of disease-suppressive soils through repeated pathogen challenge, representing ecological filtering that narrows community composition but preserves suppressive function [133]. Top-down approaches preserve ecological realism and emergent properties shaped by their native context and priority effects.

Bottom-up SynCom assemblies build microbial communities from selected isolated strains with defined traits and evaluate their collective performance under controlled conditions. This reductionist approach seeks to reveal causal relationships and emergent functions from well-characterised bacterial components [50,134] (Figure 3). This approach emphasises mechanistic inference and the tractability of the inoculant *in planta*. In this context, one promising strategy is to enrich functionally critical or keystone species, combining microorganisms with complementary plant growth promoting traits and synergistic effects that enhance community stability and jointly improve plant growth [135–137]. Metagenomics analyses have been used to identify suitable culture media and growth conditions [138,139]. The combination of culture-independent microbiome analysis with microbiome-guided isolation and cultivation strategies was used to construct a sugarcane-derived microbial consortium [140]. This consortium was applied to maize plants and was shown to modulate plant physiology, enhance drought resilience, and increase yields under drought stress by up to 3.45–3.9 fold [141].

Integrated multi-omic analysis is a robust strategy to elucidate the functional mechanisms of plant–microbiome interactions. For example, combining metagenomic and plant transcriptomic approaches enabled the functional characterisation of a tomato microbial consortium, revealing its role in modulating host gene expression related to growth regulation and defence signalling [142]. Integrating metabolomics, host plant transcriptomics, and genomic analyses helped to map how microbial exometabolites mediate microbial interactions, providing insights into the molecular mechanisms that predict the stability and coexistence of microbial consortia [143]. Alternative approaches focus on the identification of core microbiomes, taxa consistently associated with a plant across multiple environments, enabling the selection and design of stable and resilient engineered consortia, that have been proven to promote plant growth. Computational models incorporating artificial intelligence or machine learning are also changing how we rationalise the consortia selection process. These approaches use complex datasets to predict causal microbe–microbe and plant–microbiome interactions across diverse environments [144,145]. For instance, Hitch et al. [146] provided a pipeline for selecting SynComs from rumen, mouse, and human microbiomes. This pipeline identifies bacterial strains that encode key functions by using metagenomic information, and focuses on ecosystem-level functions using multiple metagenomes, rather than on an environment-specific metagenome. The consortium selection process is then refined by applying genome-scale metabolic models, providing informed *in silico* predictions of microbe–microbe interactions before experimental testing [146]. This pipeline can be translated to plant-associated communities; indeed, metabolic models were employed to select a minimal SynCom that promoted growth across soybean, maize, sorghum, and sugarcane [147].

Despite the potential of these plant-associated communities as bioinoculants, several microbial challenges should be addressed for an efficient translation from lab-controlled conditions to the field. Some of these challenges include differences in growth rates, which make it difficult to co-culture bacterial strains simultaneously, as well as variations in nutritional requirements, compatibility issues, and competition among strains. Consequently, cultivating bacterial consortia at an industrial scale remains challenging, highlighting the need to reduce the number of strains to lower costs and simplify workflows [104]. Overall, the practical application of these bacterial communities as bioinoculants requires effective formulations and delivery methods compatible with farming systems to ensure their persistence in the soil and successful plant colonisation [131,148].

## Conclusions

Altogether, beneficial plant–bacterial associations emerge from reciprocal trait compatibility: plant traits create selective niches, while microbial genetic repertoires determine colonisation success. A successful plant coloniser must be able to exploit host-derived resources, interact competitively and cooperatively within the microbial community, and establish an immune-compatible relationship with the host. These functional capacities are conserved across diverse microbial lineages, explaining the recurrent dominance of specific phyla in plant-associated bacteriomes. Many of these microbial traits are influenced by variation in plant genomes, which helps shape differences in microbiome composition and function across host genotypes. We therefore position bacterial social mechanisms as a plausible working framework for shaping plant health, rather than a definitively causal mechanism, given that not all available evidence supports causal inference. As our understanding of these determinants deepens, current approaches increasingly enable the rational design of optimised bacterial consortia as bioinoculants, opening new avenues for enhancing plant health, resilience, and productivity while also highlighting that plant-associated bacteria thrive not in isolation, but through the interconnected, village-like communities they build within plant microbiomes.

## Summary

- Plants function as ‘holobionts’, integrated units with their associated microbes, where diverse bacterial communities colonise different tissues and collectively support plant growth, health, and soil functioning.
- Bacterial community assembly is shaped by plant genetics, environmental conditions, and especially microbe–microbe interactions such as competition and cooperation, which strongly influence community structure and function.
- Many plant-associated bacteria form beneficial associations by enhancing nutrient acquisition, modulating phytohormones to shape plant development, and priming immune responses against pathogens, effects that often emerge at the community level rather than from individual species.
- Understanding how these microbial communities assemble, interact, and colonise plants enables the rational design of microbial consortia as bioinoculants, offering a promising strategy to improve crop resilience, productivity, and ecosystem sustainability.

## Open Access

Open access for this article was enabled by the participation of Consejo Superior de Investigaciones Cientificas (CSIC) in an all-inclusive Read & Publish agreement with Portland Press and the Biochemical Society under an agreement with CSIC.

## Abbreviations

HGT: horizontal gene transfer
ICEs: Integrative Conjugative Elements
JA: jasmonic acid
NCR: nodule cysteine-rich
NLR: nucleotide-binding leucine-rich repeat
PGP: plant-growth-promoting
PRRs: pattern-recognition-receptors
PSMs: plant secondary metabolites
SA: salicylic acid
SynCom: synthetic community
T6SS: Type VI Secretion System
